# Alterations of the Whole Cerebral Blood Flow in Patients With Different Total Cerebral Small Vessel Disease Burden

**DOI:** 10.3389/fnagi.2020.00175

**Published:** 2020-06-23

**Authors:** Chunyan Yu, Weizhao Lu, Jianfeng Qiu, Feng Wang, Jinglei Li, Liru Wang

**Affiliations:** ^1^Department of Radiology, Shandong First Medical University & Shandong Academy of Medical Sciences, Tai’an, China; ^2^The Second Affiliated Hospital of Shandong First Medical University, Tai’an, China; ^3^Tai’an Rongjun Hospital, Tai’an, China

**Keywords:** cerebral small vessel disease, total CSVD burden score, cerebral blood flow, MRI, arterial spin labeling

## Abstract

**Background:**

Cerebral small vessel disease (CSVD) is a common age-related vascular disease of the brain associated with slowly accumulating tissue damage. At present, total CSVD burden score is a commonly used method to evaluate the severity of the disease.

**Purpose:**

To observe whether global and regional cerebral perfusion is related to total CSVD score and to explore global and regional cerebral blood flow (CBF) changes in patients with different degrees of CSVD.

**Methods:**

We collected 130 subjects with different total burden score of CSVD (0 point: 33 subjects, 1 point: 39 subjects, 2 points: 24 subjects, 3 points: 24 subjects, 4 points: 10 subjects). Total CSVD burden score was evaluated by clinically routine sequences (T2WI, T2-FLAIR, T1WI, DWI, and SWAN sequence). Global and regional CBF were calculated and correlation analysis was used to investigate the relationship between total CSVD score and CBF of the whole brain and several brain regions.

**Results:**

The analysis results showed that there was a negative correlation between total CSVD burden score and global CBF (*r* = −0.33, *p* = 0.001). Total CSVD burden score also had moderately negative correlations with CBF of almost all the brain regions.

**Conclusion:**

CSVD is a disease that affects the whole brain. With the increase of total CSVD burden score, the global and regional CBF decreased. The CSVD total burden score could be used to evaluate the overall condition of brain perfusion.

## Introduction

Cerebral small vessel disease (CSVD) is a term used with various meanings and is used in different contexts including pathological, clinical, and neuroimaging aspects (Pantoni, 2010). CSVD is a common age-related vascular disease of the brain associated with slowly accumulating tissue damage and represents a leading cause of functional loss, disability, and cognitive decline in the elderly (Pinter et al., 2015). The clinical manifestations are diverse, patients sometimes have no obvious symptoms and sometimes have sudden stroke symptoms (Wardlaw et al., 2013a). CSVD patients with both single type of lesions and combined type of lesions have experienced cognitive impairment, dementia, depression, mobility problems, increased risk of stroke (Debette et al., 2019). The pathogenesis of CSVD is not very clear so far, and some scholars think that CSVD is related to the destruction of brain blood barrier (BBB) (Zhang et al., 2017; Thrippleton et al., 2019). Wardlaw et al. (2013a) summarized evidence suggesting that it was mainly vascular endothelial injury, which led to a series of inflammatory reactions and vascular wall self-regulating function damage. At a late stage, CSVD patients would experience luminal narrowing and occlusion, precipitating cerebral ischemia and stroke.

The most characteristic markers of CSVD under magnetic resonance imaging (MRI) are white matter hyperintensity (WMH), lacune, cerebral microbleeds (CMBs) and enlarged perivascular spaces (EPVS) (Wardlaw et al., 2013b). Staals et al. (2014) suggested that a “total CSVD score,” which combined different MRI features of CSVD in one measure, might show more value than any individual markers in representation of the overall burden of tissue damage. The score has been associated with age, male, hypertension, smoking, lacunar stroke subtype in ischemic stroke patients (Staals et al., 2014), and lower general cognitive ability (Huijts et al., 2013; Staals et al., 2015). Lau et al. (2017) validated that the total CSVD score was strongly associated with transient ischemic attack (TIA)/ischemic stroke in two large prospective cohorts. They demonstrated that patients with a higher score were at increasing risk of a recurrent ischemic stroke, and higher score could also be used to predict the subtype and prognosis. Latent variable modeling provided support for the combination of different MRI features into one overall SVD score (Staals et al., 2015).

Neuroimaging studies have demonstrated that CSVD patients experienced global alterations in brain structure and perfusion. Xu et al. (2018) successfully demonstrated that CSVD lesions disrupted both global and local structural brain networks for patient. They also showed that the overall CSVD score had insidious effect on the local clustering coefficient and nodal efficiency of the brain network (Xu et al., 2018). Lawrence et al. (2014) showed that the overall structure of the brain network in patients with CSVD was damaged, and damage degree was related to cognitive impairment. The total CSVD score and most of the individual imaging markers of CSVD were associated with the aortic pulse wave velocity (PWV) (Del Brutto et al., 2019). A study revealed that BBB permeability in the brain measured by dynamic contrast enhancement (DCE)-MRI was positively correlated with total CSVD burden (Li et al., 2018). Higher total CSVD burden in the brain was associated with lower plasma volume fraction (Li et al., 2018), and blood plasma volume was related to the cerebral blood flow (CBF) (Arba et al., 2017). However, the relationship between global CBF and total CSVD score is uncertain until now. Many studies have confirmed white matter lesions was associated with brain perfusion, high WMH load was associated with lower CBF, and white matter lesions were associated with whole cognitive function (Vernooij et al., 2008; Ban et al., 2019). However, WMH is only one of the imaging findings of CSVD, and it was not comprehensive to evaluate the severity of global brain.

Therefore, in this study, it was hypothesized that whole and regional CBF were associated with CSVD total burden score. We used the CSVD total burden score as an indicator to assess the degree of CSVD. We collected patients with different CSVD score to observe the change of global and regional CBF and to test whether there was a correlation between CBF and total CSVD burden score.

## Materials and Methods

### Participants

This cross-sectional study was approved by the Ethics Committee of Shandong First Medical University, written informed consent was obtained from all participants.

Cerebral small vessel disease patients (age from 44 to 79 years) were collected from The Second Affiliated Hospital of Shandong First Medical University with the following inclusion criteria: (1) clinical evidence of CSVD, including the following: Lacunar stroke syndrome with symptoms lasting over 24 h, occurring more than 6 months prior to the visit; or TIA lasting less than 24 h with limb weakness, hemi-sensory loss or dysarthria over 6 months previously; (2) laboratory examination: blood biochemistry indexes and liver, kidney function indexes all in normal ranges; (3) hypertension: history of hypertension (≥140/90 mmHg) (1 mmHg = 0.133 kPa) or taking antihypertensive drugs; diagnosis of hypertension without taking drugs. Hyperglycemia with the diagnostic criteria of American Diabetes Association classification and diagnosis of diabetes (Care and Suppl, 2018). Hyperlipidemia in line with the Guidelines for Prevention and Treatment of Adult Dyslipidemia in China (Geriatr, 2018). Diagnostic criteria for coronary heart disease in line with the American Heart Association; (4) signs of CSVD, such as WMH, lacune, CMBs, and EPVS using daily routine brain MRI scan.

Exclusion criteria: (1) patients with brain tumor or other systemic malignant tumors; (2) history of traumatic brain injury; (3) patients with acute massive cerebral infarction (diameter greater than 2 cm) or magnetic resonance angiography (MRA) showing artery stenosis; abnormal cerebral vascular development, such as the posterior cerebral artery of the embryonic brain; (4) liver, kidney, heart, lung, or other important organ dysfunction; (5) coma; (6) a history of craniocerebral operation; (7) cerebral hemorrhage by CT and other imaging examinations; (8) mental disorders and consciousness disorders; (9) atrial fibrillation, acute ischemic stroke caused by cardiogenic embolism.

### MRI Data Acquisition

GE’s new generation Discovery MR 750 3.0T magnetic resonance (MR) with 8-channel head coil was used in this study. All participants underwent routine MR scan and arterial spin labeling (ASL) scan. High-resolution T1-weighted image for assessing brain parenchymal volume were collected with 120 sagittal slices covering the whole brain. The scan parameters were: slice thickness = 1.2 mm, time of repetition (TR) = 8.2 ms, echo time (TE) = 3.2 ms, flip angle = 12°, field of view (FOV) = 240 × 240 mm^2^, acquisition matrix = 256 × 256, and an acquisition time of 3 min 27 s. MRA was conducted with the following parameters: TR = 23 ms, TE = 3.4 ms, FOV = 220 × 194 mm^2^, slice thickness = 1.4 mm, flip angle = 20°, vessel uniformity = 1.00. Susceptibility-weighted images (SWAN) were acquired using: TR = 45.5 ms, TE = 24.7 ms, FOV = 240 × 240 mm^2^, reconstruction resolution = 0.89 × 0.78 mm^2^, 104 contiguous slices with thickness of 3.0 mm. T2-weighted images (T2WI) were acquired using: TR = 5810ms, TE = 92ms, FOV = 240 × 240 mm^2^, slice thickness = 5 mm, acquisition matrix = 512 × 512, scanning time 1 min 4 s. T2 fluid-attenuated inversion recovery (T2 FLAIR) images were acquired with following parameters: TR = 8,500 ms, TE = 145 ms, FOV = 240 × 240 mm^2^, 20 contiguous slices with thickness of 5.0 mm, acquisition matrix of 256 × 256; scan time = 1 min 51 s. ASL parameters were as follows: sampling points on eight spirals, FOV = 220 × 220 mm^2^; reconstructed matrix = 128 × 128, TR = 4781 ms, TE = 11.1 ms, number of excitations = 3.0, slice thickness = 3.0 mm, labeling plane was positioned at the base of the cerebellum with labeling duration = 1,500 ms and post labeling delay = 1,525 ms, 90 slices covering the whole brain and acquisition time = 4 min 37 s.

### CSVD Total Burden Score Evaluation

**FIGURE 1:**
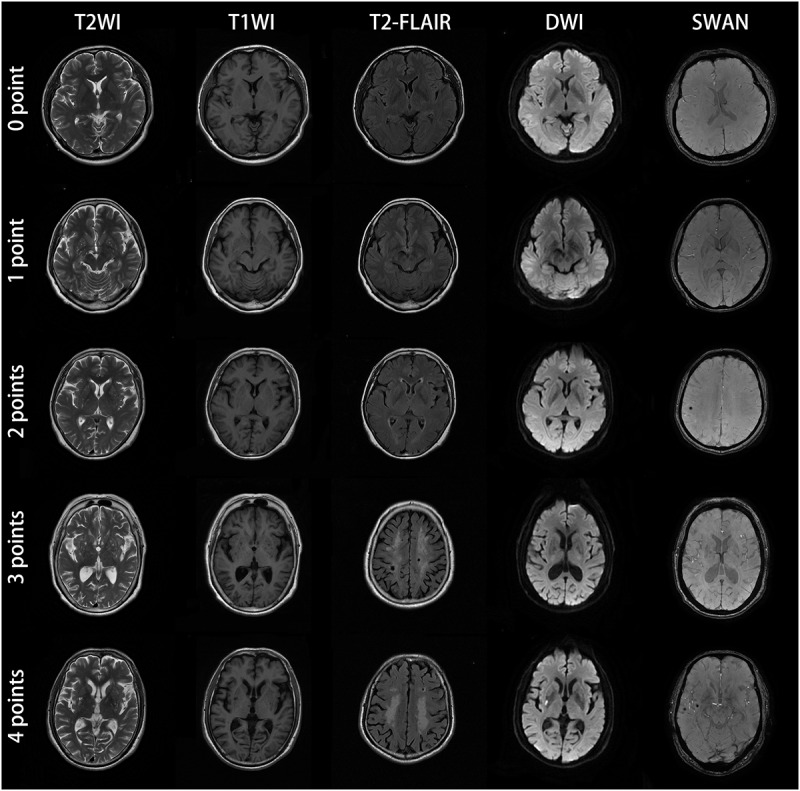
MRI images of subjects with different CSVD total burden score. The subject with score of zero point was a 57-year-old man with no abnormal lesions in MRI images. The patient with score of one point was a 61-year-old female, the bilateral basal ganglia represented punctate and linear lesions with diameter less than 3 mm, hypointensity on T1WI, hyperintensity on T2WI and low signal intensity on T2-FLAIR. The DWI and SWAN sequences showed no abnormality. The bilateral basal ganglia region coincided with EPVS (grade 2–3). For the patient with score of two points, bilateral basal ganglia showed dotted, linear cerebrospinal fluid signal shadow (diameter < 3mm), T2WI showed high signal intensity, T1WI showed low signal intensity, T2-FLAIR showed low signal intensity, which was consistent with the characteristics of EPVS. SWAN sequence of the right parietal lobe showed low signal intensity with diameter ≤ 10 mm, which conformed to the characteristics of CMBs. This CSVD patient was consistent with two points according to the criteria of total CSVD burden score. The patient with score of three points was a 60-year-old female. MRI showed EPVS (grade 2–3) in the bilateral basal ganglia, large confluent areas WMH (Fazekas 3 for DWMH) and lacunar infarction in the center of bilateral semioval center. SWAN sequences showed no abnormality. The patient with CSVD score of four points was a 62-year-old male. There were EPVS (grade 2–3) in the bilateral basal ganglia, large fused WMH (Fazekas 3 for DWMH) in the center of the bilateral semiovale center, acute lacunar infarction with high signal intensity in the right basal ganglia region in DWI image, and CMBs in the right temporal lobe. All the four kinds of imaging markers appeared in this patient.

Lacunes, WMH, CMBs, and EPVS have been identified as MRI markers of CSVD (Wardlaw et al., 2013b). Table 1 shows detailed evaluation criteria of total CSVD burden score. For lacunes, we identified a symptomatic lacunar infarct on T2WI. Periventricular and deep WMH were rated on the Fazekas scale using FLAIR and T2WI sequences (Fazekas et al., 1987). EPVS have been widely considered as a feature of CSVD (Doubal et al., 2010). EPVS were defined as clear boundary, round, oval or linear sign, generally <3 mm in diameter, T2WI high signal, T1WI low signal and FLAIR low signal, similar to cerebrospinal fluid-like signal (Yang et al., 2002). CMBs were defined as small areas (2–10 mm in diameter) of signal void with associated blooming in GRE or SWAN sequence (Tsivgoulis et al., 2016). One accumulative score for each of the four imaging marks: lacunes, severe WMH (defined as Fazekas score 2–3 for DWMH or 3 for PVWMH), EPVS (grade 2–3) and CMBs were rated by two experienced radiologists who have worked for 10 years. Negotiation were conducted when the results of measurement were inconsistent. The score varies from 0 to 4. According to the results of the previous studies (Lau et al., 2017; Li et al., 2019), we only calculated the lesions of EPVS in the basal ganglia, and did not include the semi-oval center. Figure 1 demonstrates MRI images of subjects with different total CSVD burden score.

**TABLE 1:** Show of total CSVD burden score.

**Lesions classification**	**Definition and grades**		**Number or degree**	**Total CSVD burden score**
Lacunes	We identified a symptomatic lacunar infarct as circular or oval hyperintense lesions <20 mm on T2WI with corresponding hypointense lesions with a hyperintense rim on FLAIR or hyperintense on DWI		0	0
			≥1	1
WMH	Periventricular white matter hyperintensity (PVWMH) and deep white matter hyperintensity (DWMH) were rated on the Fazekas scale using FLAIR and T2WI sequences, periventricular WMH	(PVWMH) was graded as: 0 = absence, 1 = caps or pencil-thin lining, 2 = smooth halo, and 3 = irregular PVWMH extending into the deep white matter. (DWMH) were rated as: 0 = absence, 1 = punctate foci, 2 = beginning confluence of foci, and 3 = large confluent areas	<Fazekas score 3 for PVWMH or 2–3 for DWMH	0
			≥Fazekas score 3 for PVWMH or 2–3 for DWMH	1
CMBs	CMBs were defined as small areas (2–10 mm in diameter) of signal void with associated blooming in GRE or SWAN sequence		0	0
			≥1	1
EPVS	EPVS were defined as clear boundary, round, oval or linear sign, generally <3 mm in diameter, T2WI high signal, T1WI low signal and FLAIR low signal, similar to cerebrospinal fluid-like signal	Grade: 0 = no EPVS, 1 ≤ 10 EPVS, 2 = 11–20 EPVS, 3 = 21–40 EPVS, and 4 = more than 40 EPVS	Grade 0–1	0
			Grade 2–3	1

### Extraction of Cerebral Blood Flow

**FIGURE 2:**
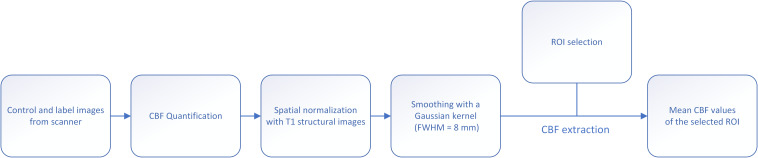
Flow chart of imaging preprocessing, segmentation and CBF extraction.

The outline of data processing and CBF extraction is given in Figure 2. The CBF map was calculated using the Functool software in the MRI server. A two-step normalization of CBF map was conducted: T1 image was firstly registered to CBF map for each subject using affine transformation, followed by a non-linear registration of T1 image to the Montreal Neurological Institute (MNI) template. At last, the transformation is applied to the CBF map. After normalization, the CBF map was smoothed with a Gaussian kernel of 8 mm FWHM.

Regions of interest (ROI) were generated using WFU pickatlas, and CBF values of each ROI were extracted. We included the following ROIs in the study: frontal cortex, frontal white matter, parietal cortex, parietal white matter, temporal cortex, temporal white matter, occipital cortex, occipital white matter, insular lobe, whole cortex, whole white matter, basal ganglia, thalamus, corpus callosum, infratentorial, whole brain, and extract the mean CBF values of the corresponding brain regions.

### Statistical Analyses

One-way ANOVA was used to assess age among groups with different total CSVD burden scores. Chi square test was used to access differences in gender, prevalence (hypertension, hyperlipidemia, diabetes, and coronary heart disease), bad habits (ever smoking history and ever drinking history) among different groups. Ordered logistic regression analysis was performed to further investigate the relationship between risk factors and CSVD severity. In the ordered Logistic regression analysis, we used eight clinical risk factors (age, female, hypertension, diabetes, hyperlipidemia, coronary heart disease, ever smoking, and ever alcohol) as input variables and CSVD total burden score as dependent variable. Pearson correlation analysis was performed between age and CBF of whole brain and ROIs. Partial correlation (controlled for age) analysis was used to analyze the correlation between global CBF, regional CBF and total CSVD score. All statistical analyses were performed using SPSS 24.0 with significance set at *p* < 0.05. Correlation analyses were corrected by Bonferroni method to control false positives, *p* < 0.003 was considered statistical significant after correction.

## Results

### Demographic Information

A total of 130 subjects were collected, including 33 cases of 0 point, 39 cases of 1 point, 24 cases of 2 points, 24 cases of 3 points, and 10 cases of 4 points. There were no MRI markers of CSVD in 0-point subjects, but there might be some risk factors. The demographic and clinical information are shown in Table 2. There was a significant difference (*F* = 3.17, *p* = 0.016) in age among different CSVD score groups, there was no significant difference (χ^2^ = 0.965, *p* = 0.915) in gender among the groups. There were no significant differences in the prevalence of hypertension (χ^2^ = 8.94, *p* = 0.062), diabetes (χ^2^ = 4.71, *p* = 0.319), hyperlipidemia (χ^2^ = 1.65, *p* = 0.800), heart disease (χ^2^ = 4.07, *p* = 0.396), and ever drinking alcohol (χ^2^ = 8.37, *p* = 0.079) among the groups. There was a significant difference in smoking history among the groups (χ^2^ = 14.31, *p* = 0.006).

**TABLE 2:** Baseline clinical and demographic data.

	**0 point (*n* = 33)**	**1 point (*n* = 39)**	**2 points (*n* = 24)**	**3 points (*n* = 24)**	**4 points (*n* = 10)**	***P*-value**
Age (mean ± SD)	59.8 ± 8.1	63.1 ± 6.4	65.9 ± 9.3	65.8 ± 9.2	66.5 ± 7.2	0.016
Female/Male	18/15	20/19	10/14	12/12	5/5	0.915
Hypertension (%)	39.4	51.3	62.5	70.8	80.0	0.062
Diabetes (%)	15.2	17.9	33.3	33.3	30.0	0.319
Hyperlipidemia (%)	24.2	20.5	33.3	29.2	20.0	0.800
Coronary heart disease (%)	15.2	20.5	33.3	33.3	20.0	0.396
Ever smoking^⋇^(%)	12.1	15.4	20.8	45.8	50.0	0.006
Ever alcohol^⋇^ (%)	15.2	30.8	33.3	37.5	60.0	0.079

*^⋇^Ever smoking (alcohol) refers to a history of drinking (alcohol) before, whether or not it is still drinking does not take into consideration.*

### Ordered Logistic Regression Analysis of the Risk Factors for CSVD

Ordered logistic regression results are given in Table 3. Results showed that only age (B = 0.006, *p* = 0.003) and smoking history (B = 0.98, *p* = 0.029) could significantly affect the severity of CSVD, other variables were not statistically significant (*p* > 0.05). It was noteworthy that the Exp (B) of age and smoking history were 1.07 and 2.67, respectively, indicating that smoking history had a larger impact on the severity of the disease than age.

**TABLE 3:** The result of logistic regression analysis.

	**B**	**SE**	**Wals**	**Sig**	**Exp(B)**	**95% CI of Exp (B)**	
						**Lower limit**	**Upper limit**
Age	0.006	0.02	8.92	**0.003**	1.07	1.02	1.11
Female	0.32	0.41	0.60	0.439	1.37	0.62	3.05
Hypertension (Yes)	0.63	0.37	2.89	0.089	1.88	0.91	3.88
Diabetes (Yes)	0.10	0.40	0.06	0.809	1.10	0.50	2.41
Hyperlipidemia (Yes)	−0.11	0.38	0.09	0.762	0.89	0.43	1.87
Coronary heart disease (Yes)	0	0.40	0	0.994	1.00	0.46	2.19
Ever smoking	0.98	0.45	4.77	**0.029**	2.67	1.11	6.44
Ever alcohol	0.51	0.47	1.14	0.286	1.66	0.65	4.20

*B, regression coefficient; SE, standard error; Wals, wald test; Sig, significance; Exp(B), the exponent of B or relative risk. Significant differences are indicated in bold.*

### Age and Whole Brain CBF

As shown in Table 4, there was moderately negative correlation between age and CBF in the whole brain and in all brain regions (*p* < 0.003, Bonferroni corrected). The correlation coefficient between age and CBF in the frontal cortex was the highest (*r* = −0.44, *p* < 0.001), correlation between age and CBF in the basal ganglia was the weakest (*r* = −0.28, *p* = 0.001).

**TABLE 4:** Correlation between age and CBF in whole brain and regional brain.

	**Frontal cortex**	**Frontal WM^⋇^**	**Parietal cortex**	**Parietal WM**	**Temporal cortex**	**Temporal WM**
*R* value	−0.44	−0.35	−0.44	−0.39	−0.41	−0.34
*P* value	<0.001	<0.001	<0.001	<0.001	<0.001	<0.001

	**Occipital cortex**	**Occipital WM**	**Insular lobe**	**Whole cortex**	**Whole WM**	

*R* value	−0.41	−0.39	−0.33	−0.43	v0.37	
*P* value	<0.001	<0.001	<0.001	<0.001	<0.001	

	Basal ganglia*	**Thalamus**	**Corpus callosum**	Infratentorial**	**Whole brain**	

*R* value	−0.28	−0.40	−0.43	−0.42	−0.42	
*P* value	0.001	<0.001	<0.001	<0.001	<0.001	

^⋇^WM, white matter. *Basal ganglia including the caudate nucleus, lenticular nucleus, amygdala, claustrum, internal capsule, and external capsule. **Infratentorial including the brainstem (mesencephalon, cerebelli, and medulla oblongata) and cerebellum.

### CBF and Total CSVD Burden Score

**FIGURE 3:**
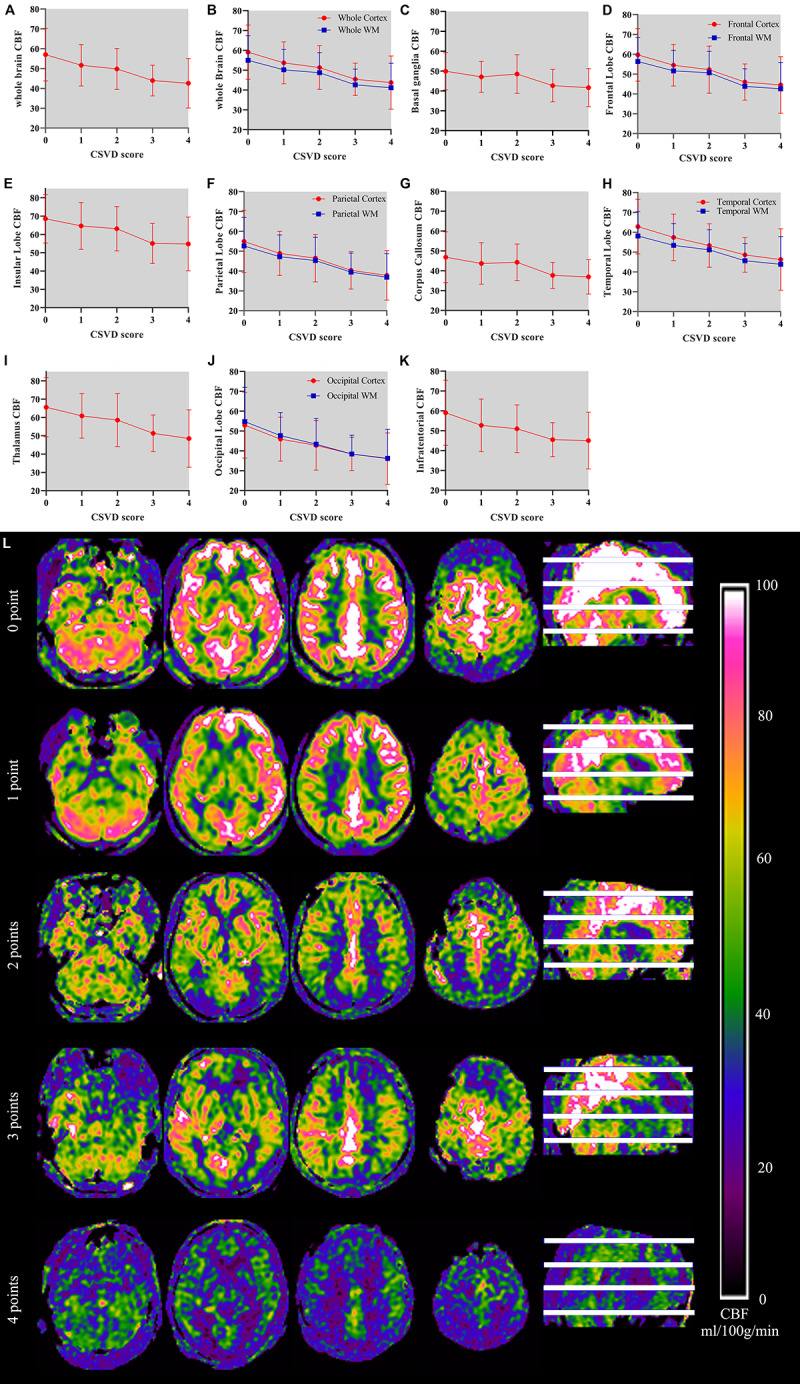
**(A–K)** Demonstrates relationship between CSVD total burden score and CBF in the whole brain and several brain regions and CBF maps of subjects with different CSVD total burden scores. The horizontal axis on the picture represents CSVD total burden score and the vertical axis represents the CBF values (unit: ml/100 g/min) of the corresponding brain region. In **(B)**, **(D)**, **(F)**, **(H)**, and **(J)**, red line represents CBF of the cortex and blue represents CBF of the white matter. Error bar represents mean and standard deviation. **(L)** shows CBF maps of five subjects with CSVD total burden from 0 to 4.

Table 5 shows the perfusion values of whole brain and several brain regions among five groups with different CSVD score. Among different regions, CBF value in the insular lobe in the group with CSVD total burden score of 0 point was the highest (68.56 ± 13.26 ml/100 g/min), and CBF value in the occipital cortex in the group with CSVD score of four points was the lowest (36.06 ± 12.99 ml/100 g/min) (see Table 5). We plotted CBF values for several brain regions across groups with different CSVD total burden scores (Figures 3A–K), and showed brain CBF maps for subjects with different CSVD total burden scores (Figure 3L). It could be seen that with the increase of the CSVD score, CBF values of almost all of the brain regions showed a decreased trend. In addition, CBF of the corpus callosum and basal ganglia decreased slowly, the other regions decreased slightly faster. Gray matter CBF values in most brain regions were higher than that of the white matter except the occipital lobe across all groups (see Figure 3). The correlation analysis results between CBF (controlled for age) in whole brain and regional brain and CSVD severity are shown in Table 6. Whole brain CBF was negatively correlated with CSVD score (*r* = −0.33, *p* < 0.001). CBF in most brain regions were significantly correlated with CSVD scores (*p* < 0.003, Bonferroni corrected), except for the basal ganglia (*r* = −0.22, *p* = 0.013) and corpus callosum (*r* = −0.21, *p* = 0.016). Correlation between CBF in the infratentorial and CSVD total burden score was the weakest (*r* = −0.26, *p* = 0.003), CBF in the temporal cortex (*r* = −0.36, *p* < 0.001) and occipital white matter (*r* = −0.36, *p* < 0.001) had the strongest correlation with CSVD total burden score (see Table 6).

**TABLE 5:** Perfusion of whole brain and various brain regions.

	**0 point (*n* = 33)**	**1 point (*n* = 38)**	**2 point (*n* = 24)**	**3 point (*n* = 24)**	**4 point (*n* = 10)**
Frontal cortex	59.71 ± 13.26	54.46 ± 10.51	52.21 ± 11.90	46.00 ± 9.18	44.50 ± 14.29
Frontal WM^⋇^	56.36 ± 12.04	51.64 ± 10.37	50.65 ± 10.76	43.82 ± 8.80	42.57 ± 13.30
Parietal cortex	54.90 ± 15.70	48.89 ± 11.13	40.37 ± 12.02	40.37 ± 9.48	37.81 ± 12.50
Parietal WM	52.66 ± 14.35	47.21 ± 10.99	45.27 ± 11.68	39.43 ± 9.61	36.90 ± 11.85
Temporal cortex	62.87 ± 13.78	57.43 ± 11.77	53.31 ± 11.00	48.58 ± 8.75	46.25 ± 15.52
Temporal WM	58.14 ± 12.23	53.41 ± 10.94	51.10 ± 10.19	45.61 ± 8.66	43.86 ± 14.01
Occipital cortex	52.92 ± 16.56	45.91 ± 11.10	42.78 ± 12.48	38.42 ± 8.37	36.06 ± 12.99
Occipital WM	54.75 ± 17.22	47.73 ± 11.64	43.39 ± 13.03	38.41 ± 9.51	36.25 ± 14.54
Insular lobe	68.56 ± 13.26	64.64 ± 12.84	63.11 ± 12.11	55.10 ± 10.93	54.80 ± 14.71
Whole cortex	59.12 ± 13.67	53.69 ± 10.58	51.33 ± 11.04	45.43 ± 8.15	43.78 ± 13.43
Whole WM	54.99 ± 12.39	50.21 ± 10.18	48.71 ± 10.16	42.63 ± 7.95	41.18 ± 12.35
Corpus callosum	46.80 ± 12.82	43.67 ± 10.49	44.27 ± 9.23	37.68 ± 6.56	36.88 ± 8.72
Basal ganglia*	49.96 ± 9.41	47.10 ± 7.81	48.53 ± 9.76	42.67 ± 8.19	41.66 ± 9.61
Thalamus	65.60 ± 16.14	60.89 ± 12.19	58.55 ± 14.45	51.35 ± 10.00	48.48 ± 15.71
Infratentorial**	59.02 ± 16.43	52.67 ± 13.27	50.97 ± 12.03	45.46 ± 8.61	45.02 ± 14.35
Whole brain	56.10 ± 13.30	51.67 ± 10.47	49.82 ± 10.33	43.94 ± 7.77	42.61 ± 12.52

Unit: ml/(100 g min). ^⋇^WM, white matter; *Basal ganglia including the caudate nucleus, lenticular nucleus, amygdala, claustrum, internal capsule, and external capsule. **Infratentorial including the brainstem (mesencephalon, cerebelli, and medulla oblongata) and cerebellum.

**TABLE 6:** Correlation between total CSVD score and whole brain and regional brain.

	**Frontal cortex**	**Frontal WM^⋇^**	**Parietal cortex**	**Parietal WM**	**Temporal cortex**	**Temporal WM**
*R* value	−0.33	−0.33	−0.34	−0.32	−0.36	−0.34
*P* value	<0.001	<0.001	<0.001	<0.001	<0.001	<0.001

	**Occipital cortex**		**Occipital WM**	**Insular lobe**	**Whole cortex**	**Whole WM**

*R* value	−0.33		−0.36	−0.30	−0.34	−0.33
*P* value	<0.001		<0.001	0.001	<0.001	<0.001

	Basal ganglia*		**Thalamus**	**Corpus callosum**	Infratentorial**	**Whole brain**

*R* value	−0.22		−0.31	−0.21	−0.26	−0.33
*P* value	0.013		<0.001	0.016	0.003	<0.001

^⋇^WM: White matter; *Basal ganglia including the caudate nucleus, lenticular nucleus, amygdala, claustrum, internal capsule, external capsule; **Infratentorial including the brainstem (mesencephalon, cerebelli, medulla oblongata) and cerebellum.

## Discussion

Small vessel disease was regarded as a dynamic, whole-brain disorder (Wardlaw et al., 2019). Previous research showed that there was a correlation between microinfarction of CSVD and global CBF, and found microinfarction could lead to decreased CBF throughout the anterior circulation rather than being confined to the area around them (Ferro et al., 2019). The presence of microbleeds was also reported to be related to reduced brain perfusion (Gregg et al., 2015). In the present study, we aimed to find the relationship between brain perfusion and disease severity by performing correlation analysis between brain CBF values and CSVD total burden score. Results demonstrated that CSVD total burden score had a moderately negative correlation with CBF of the whole brain. With the increase of CSVD total burden, the CBF of whole brain and several brain regions decreased. Our results confirmed that CSVD was a disease accumulating whole brain damage. As the disease progressed, the blood flow in the whole brain and most brain regions decreased and the damage to the whole brain was more aggravated, no matter where the lesion was.

The negative association between CSVD severity and CBF could be explained by several mechanisms: The main pathological mechanism is endothelial dysfunction, and impaired self-regulating function, especially vasodilation and contraction, which eventually lead to the instability of blood supply of brain tissue (Bailey et al., 2012; Wardlaw et al., 2013a). Recent evidence have shown that endothelial dysfunction could significantly contribute to dysregulated capillary flow (Erdener and Dalkara, 2019). Another pathogenic mechanism was BBB disruption (Caplan, 2015). One of the important reasons we hold for this might be that all four imaging markers could destroy BBB. Both BBB permeability and CBF regulation are functional elements controlled in the neurovascular unit (NVU) (Stanimirovic and Friedman, 2012). NVU was composed of neurons, endothelial cells, astrocytes, pericytes, and vascular smooth muscle cells (Iadecola, 2017). Destruction of BBB could lead to decreased CBF (Cucullo et al., 2011). The link between BBB impairment and decreased CBF suggests that the defect of one functional element of the NVU can affect other NVU element (Wong et al., 2019). The disruption of BBB, whether due to the change of basal plate and the injury of endothelial cells or astrocytes, is a common term of many neurodegenerative diseases with microcirculation pathology (Erdener and Dalkara, 2019). Another hypothesis assumed that CSVD and CBF were associated with chronic hypoperfusion or impaired cerebrovascular reactivity (Pantoni, 2010). When the activity of the brain decreased, the CBF in this area decreased. In other words, lower brain activity requires lower energy (LeMaistre Stobart et al., 2013).

In terms of the relationship between regional CBF and CSVD total burden score, a major finding was that CBF in most brain regions was negatively correlated with the disease severity, and the relationship differed between the cerebral lobes, which was consistent with the previous findings (Hatada et al., 2019). There was evidence derived from previous studies suggesting that CBF was reduced in CSVD, particularly in subcortical white matter (Bernbaum et al., 2015; Arba et al., 2017). However, our results showed that CSVD accumulated not only in white matter, but also in cortex and other areas. A study confirmed that high WMH predicted subsequent lower CBF (Van Der Veen et al., 2015). Ten Dam et al. (2007) demonstrated in 390 cases that a decline in global CBF over 2.75 years was associated with a progression in periventricular WMH but not in deep WMH. A systematic review showed that CBF was negatively correlated with the severity of WMH, and white matter lesions led to a decreased perfusion in global brain and cortical structure rather than reverse (Shi et al., 2016, 2020). Although perfusion may decrease at the lesion sites, particular for WMH, the consistent finding for the different manifestations of CSVD is that they predominantly associate with reduced CBF in the whole brain (Ferro et al., 2019).

Although our study demonstrated the perfusion of whole brain and several brain regions decreased with the increase of CSVD score, this phenomenon did not exist in the basal ganglia and corpus callosum. The reason may be due to the fact that the basal ganglia is a subcortical structure which have connections with the cerebral cortex, thalamus, and other brain areas. Blood supply mechanism of the basal ganglia is complicated (Cakir, 2019). As the largest subcortical commissural fiber, the corpus callosum plays an important role in cerebral functions and has abundant blood supply from bilateral circulation (Zhang et al., 2019). Therefore, CBF in the basal ganglia and corpus callosum are complicated and need to be further studied.

Our results also showed that CBF in the whole brain and all brain regions decreased with age. In addition, age and smoking history were significant risk factors contributing to the disease progression identified by the ordered logistic regression analysis. Age- and gender-related alterations in brain CBF have been found in the health (Chen et al., 2011). Ambarki et al. (2015) have shown that women have higher CBF values than men. Several studies have also found evidence of decreased cerebral perfusion with aging, using different imaging techniques (Chen et al., 2011; Liu et al., 2011). Because aging causes general brain atrophy and cortical thinning, which may increase the partial volume effects (Slosman et al., 2001), and may disrupt the relationship between CBF and intrinsic functional connectivity via neurovascular dysregulation (Galiano et al., 2019). In terms of smoking, previous studies have shown smoking habit was associated with cognitive decline, lower cortical volumes and higher WMH volumes (Cox et al., 2019; Turon et al., 2020). Smoking was associated with ischemic CSVD as revealed by the state-of-the-art study (Larsson et al., 2019). Hara et al. (2019) have found a synergistic effect of hypertension and smoking on the total CSVD burden score. In line with previous findings, the present results may reveal that severity and progression of CSVD may be affected by classical risk factors such as aging and smoking, and control of these risk factors is strongly advised for all CSVD patients. However, other risk factors such as hypertension, diabetes, etc. were not significantly related to CSVD total score in the present study, which may be due to the fact that the relatively medium sample size and binary variables representing these risk factors weakened the statistical power.

Several issues limited the interpretation of the current results. Firstly, this was a medium sample study. We need a larger sample to further confirm the reliability of the results. Secondly, we only collected binary variables of the risk factors, such as hypertension and hyperglycemia, which may affect the statistical analysis. Thirdly, with regard to age, there were differences in age among different groups in our study, although we controlled age in the correlation analysis between CSVD score and CBF, we could not absolutely rule out the influence of age on the experimental results. Lastly, with regard to the selection of ROIs, we selected ROIs according to the structure of the cerebral lobes, but we have not considered whether other methods of ROI selection (such as selecting ROIs based on cognitive functions) are more meaningful.

## Conclusion

In this study, we have investigated the relationship between CSVD score and cerebral perfusion. Our study confirmed that the CSVD total burden score was related to global CBF and CSVD was a cumulative whole brain disease. With the aggravation of CSVD disease, the CBF of whole brain and most brain regions would decrease. Therefore, CSVD score could be used to evaluate the overall condition of whole brain perfusion. The results also revealed that certain risk factors were related to CSVD severity and should be controlled for CSVD patients.

## Data Availability Statement

The raw data supporting the conclusions of this article will be made available by the authors, without undue reservation.

## Ethics Statement

The studies involving human participants were reviewed and approved by Ethics Committee of Shandong First Medical University. The patients/participants provided their written informed consent to participate in this study. Written informed consent was obtained from the individual(s) for the publication of any potentially identifiable images or data included in this article.

## Author Contributions

WL provided management of image analysis, follow-up lesion segmentation, and edited manuscript. JQ provided co-registration of images and help with image analysis. FW provided conceptualization of this study and designed study. CY provided acquisition and analysis data, writing of manuscript. LW and JL provided substantial contributions to the acquisition of data for the work.

## Conflict of Interest

The authors declare that the research was conducted in the absence of any commercial or financial relationships that could be construed as a potential conflict of interest.
